# Thickness‐ and Wavelength‐Dependent Nonlinear Optical Absorption in 2D Layered MXene Films

**DOI:** 10.1002/smsc.202400179

**Published:** 2024-06-13

**Authors:** Di Jin, Wenbo Liu, Linnan Jia, Yuning Zhang, Junkai Hu, Houssein El Dirani, Sébastien Kerdiles, Corrado Sciancalepore, Pierre Demongodin, Christian Grillet, Christelle Monat, Duan Huang, Jiayang Wu, Baohua Jia, David J. Moss

**Affiliations:** ^1^ Optical Sciences Centre Swinburne University of Technology Hawthorn 3122 Victoria Australia; ^2^ School of Automation Central South University Changsha 410083 China; ^3^ Centre for Atomaterials and Nanomanufacturing (CAN), School of Science RMIT University Melbourne 3000 Victoria Australia; ^4^ School of Physics Peking University Haidian District Beijing 100871 China; ^5^ STMicroelectronics Crolles Cedex 38926 France; ^6^ CEA‐LETI, Minatec, Optics and Photonics Division Grenoble 38054 France; ^7^ Soitec SA Bernin 38190 France; ^8^ Ecole Centrale de Lyon Université de Lyon Ecully 69130 France; ^9^ ARC Centre of Excellence in Optical Microcombs for Breakthrough Science (COMBS) Melbourne 3000 Victoria Australia; ^10^ School of Eletronic Information Central South University Changsha 410083 China; ^11^ Hefei National Laboratory Hefei 230088 China; ^12^ The Australian Research Council (ARC) Industrial Transformation Training Centre in Surface Engineering for Advanced Materials (SEAM) RMIT University Melbourne 3000 Victoria Australia

**Keywords:** 2D materials, MXenes, nonlinear optics, on‐chip integrations, *Z*‐scan technique

## Abstract

As a rapidly expanding family of 2D materials, MXenes have recently gained considerable attention. Herein, by developing a coating method that enables transfer‐free and layer‐by‐layer film coating, the nonlinear optical absorption (NOA) of Ti_3_C_2_T_*x*_ MXene films is investigated. Using the *Z*‐scan technique, the NOA of the MXene films is characterized at ≈800 nm. The results show that there is a strong and layer‐dependent NOA behavior, transitioning from reverse saturable absorption (RSA) to saturable absorption (SA) as the layer number increases from 5 to 30. Notably, the nonlinear absorption coefficient *β* changes significantly from ≈7.13 × 10^2^ cm GW^−1^ to ≈−2.69 × 10^2^ cm GW^−1^ within this range. The power‐dependent NOA of the MXene films is also characterized, and a decreasing trend in *β* is observed for increasing laser intensity. Finally, the NOA of 2D MXene films at ≈1550 nm is characterized by integrating them onto silicon nitride waveguides, where an SA behavior is observed for the films including 5 and 10 layers of MXene, in contrast to the RSA observed at ≈800 nm. These results reveal intriguing nonlinear optical properties of 2D MXene films, highlighting their versatility and potential for implementing high‐performance nonlinear photonic devices.

## Graphical abstract

Nonlinear optical absorption (NOA) behaviors of 2D layered MXene films are characterized using both the *Z*‐scan technique and by integrating them onto silicon nitride photonic waveguides. Experimental results show that the NOA varies significantly as the light wavelength and film thickness changes, highlighting the versatility and potential of 2D MXene films for implementing high‐performance nonlinear photonic devices.

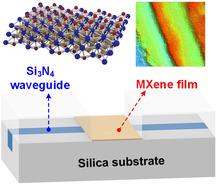

## 1 Introduction

Nonlinear photonic devices offer a powerful solution for realizing ultrafast information processing through all‐optical signal processing, which surpasses the capabilities of electronic processing by providing speeds that are several orders of magnitude higher.^[^ ^1^, ^2^, ^3^ ^]^ As fundamental building blocks for implementing nonlinear photonic devices, advanced optical materials with excellent nonlinear optical properties have been extensively investigated.^[^ ^4^, ^5^ ^]^ Recently, there has been increasing interest in the nonlinear optical properties of 2D materials,^[^ ^6^, ^7^, ^8^, ^9^, ^10^ ^]^ which have atomically thin structures and exhibit many remarkable properties that are much superior to those of conventional bulk materials.^[^ ^11^, ^12^, ^13^ ^]^ A variety of 2D materials, such as graphene,^[^ ^14^, ^15^ ^]^ graphene oxide (GO),^[^ ^16^, ^17^ ^]^ transition metal dichalcogenides (TMDCs),^[^ ^8^, ^18^ ^]^ black phosphorus (BP),^[^ ^19^, ^20^ ^]^ perovskite,^[^ ^9^, ^21^ ^]^ and MXene,^[^ ^22^, ^23^, ^24^ ^]^ have been investigated, exhibiting attractive properties such as strong saturable absorption (SA) or reverse saturable absorption (RSA), ultrahigh second‐ or third‐order optical nonlinearity, significant material anisotropy, and broadband response. These properties have enabled the development of various nonlinear photonic devices for diverse applications, such as mode‐locking lasers,^[^ ^25^, ^26^ ^]^ all‐optical modulators,^[^ ^27^, ^28^ ^]^ polarization‐dependent all‐optical switches,^[^ ^20^, ^29^ ^]^ and nonlinear optical generation and processing.^[^ ^30^, ^31^, ^32^ ^]^

As a new category of 2D materials that has received significant attention in recent years, MXenes have shown many exceptional mechanical, thermal, electrical, and optical properties.^[^ ^33^, ^34^ ^]^ For example, the rich surface groups and flexible layer spacing in MXene make it a highly effective photocatalyst.^[^ ^35^, ^36^ ^]^ Moreover, MXenes have the capability to absorb near‐infrared radiation, resulting in elevated photothermal conversion efficacy.^[^ ^37^, ^38^ ^]^ The exceptional plasma characteristics have also underpinned the realization of plasma photodetectors (PDs) and surface‐enhanced Raman spectroscopy.^[^ ^39^, ^40^ ^]^ Recently,^[^ ^22^, ^23^ ^]^ it has been reported that MXenes exhibit significant nonlinear optical absorption (NOA) that is two orders of magnitude higher than BP^[^ ^41^ ^]^ and molybdenum disulfide (MoS_2_).^[^ ^42^ ^]^ However, no detailed characterization has been conducted to examine how the NOA properties are influenced by the film thickness.

In this article, we prepare layered Ti_3_C_2_T_*x*_ films via a solution‐based method that yields transfer‐free and layer‐by‐layer film coating, which allows us to investigate the layer‐dependent NOA of the 2D MXene films that has not been characterized previously. We utilize the *Z*‐scan technique to measure the NOA of the MXene films at ≈800 nm, and the results reveal a strong layer‐dependent NOA behavior, transitioning from RSA to SA as the layer number increases from 5 to 30. Remarkably, the nonlinear absorption coefficient *β* undergoes a considerable change within this range, varying from ≈7.13 × 10^2^ cm GW^−1^ to ≈−2.69 × 10^2^ cm GW^−1^. In addition, we characterize the nonlinear response of the MXene films at varying incident laser intensities and observe a decreasing trend in *β* as the laser intensity increases. Finally, we integrate 2D MXene films onto silicon nitride (Si_3_N_4_) waveguides to characterize the NOA at ≈1550 nm. Experimental results show that the 5‐layer and 10‐layer MXene films exhibits wavelength‐dependent behavior, transitioning from RSA to SA as the wavelength changes from ≈800 to ≈1550 nm. These results reveal interesting insights about the evolution of the nonlinear optical properties of 2D MXenes as the film thickness increases. Furthermore, our MXene film coating method is highly compatible with integrated photonic devices. All of these pave the way for their applications in high‐performance nonlinear photonic devices.

## 2 Experimental Section

**Figure 1:**
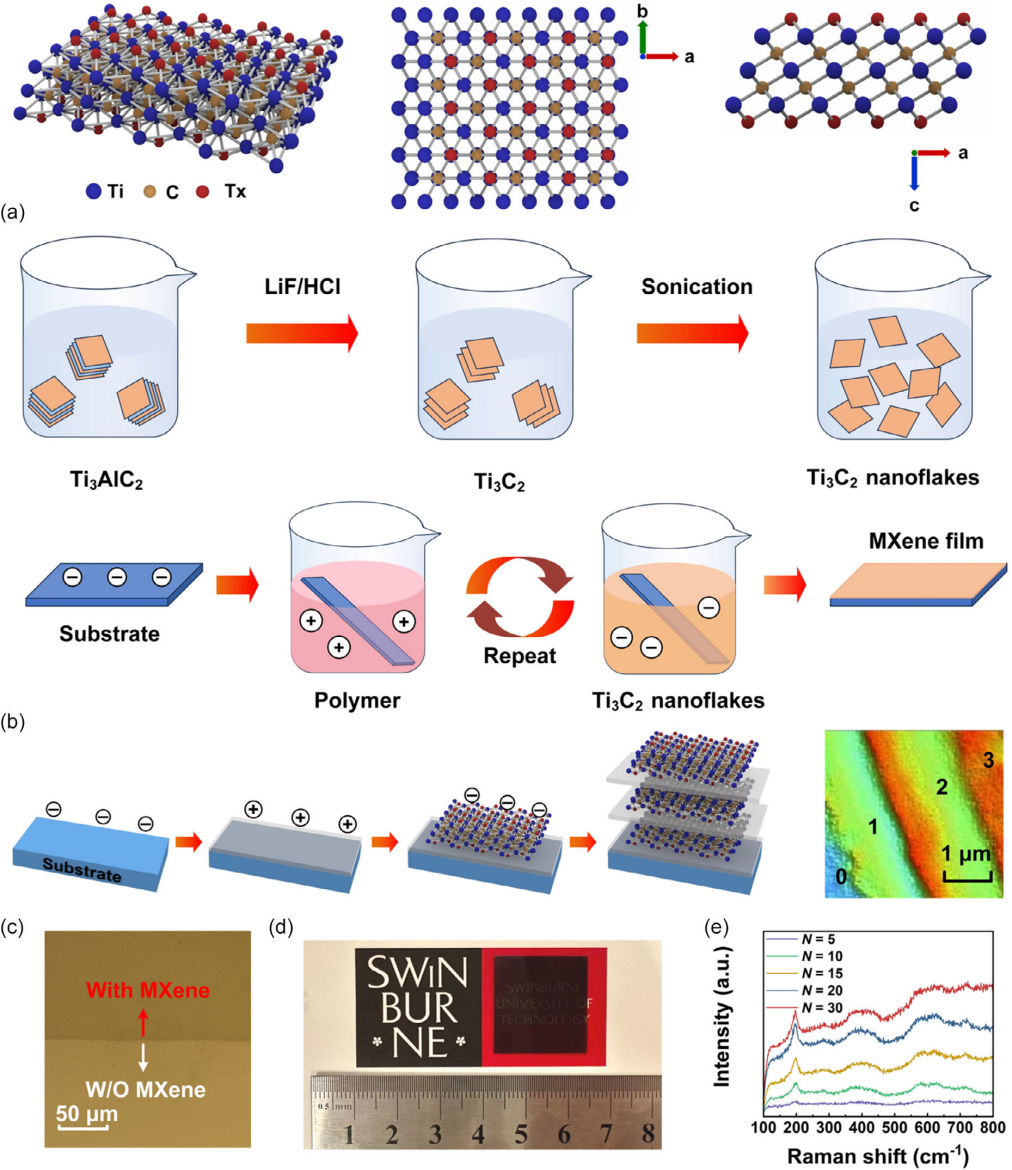
a) Schematic atomic structure of MXene consisting of layered Ti_3_C_2_. b) Schematic illustration of process flow used to fabricate MXene films via self‐assembly. The top row depicts the process flow for preparing a solution containing 2D MXene nanoflakes. The middle row illustrates the process flow for coating the MXene film on a substrate through self‐assembly. The bottom row illustrates the progression of the sample during the self‐assembly process (left) and the 3D profilometer image of the prepared MXene films with layered structure (right). In the 3D profilometer image, the numbers 1−3 refer to the number of MXene layers for that part of the image. The number 0 refers to the silica substrate. c) Microscope image of a silica substrate without MXene and with monolayer MXene. d) Photograph of a 10‐layer MXene film coated on a silica substrate. The ruler unit is in centimeters. e) Raman spectra for the prepared MXene films with different layer numbers *N* = 5, 10, 15, 20, and 30.

**Figure** **1**a illustrates the atomic structure of Ti_3_C_2_T_x_, which is an important member of the MXene family that has been widely studied.^[^ ^43^ ^]^ The basic structure of Ti_3_C_2_T_*x*_ is composed of alternating stacks of Ti and C atoms, and thick Ti_3_C_2_T_*x*_ films are made up of Ti_3_C_2_ layers that are divided into groups containing randomly distributed elements such as ‐O, ‐OH, and ‐F. The surface functional groups of MXene materials play a crucial role in determining their properties.^[^ ^44^ ^]^ For example, the hydroxylated and fluorinated terminations are more transparent, while the oxygen termination increases both absorption and reflectance at visible wavelengths.^[^ ^45^ ^]^ In addition, upon absorbing light, the energy of the photon in MXene film could transform into lattice motion inside the atomic structure, leading to the generation of phonons as well as the increase of the film temperature.^[^ ^37^ ^]^

We fabricated MXene films on dielectric substrates through self‐assembly of MXene nanoflakes in a solution by electrostatic attachment. Figure 1b illustrates the fabrication process flow. Before film coating, a MXene solution and a polymer solution were prepared. The former contained negatively charged 2D MXene nanoflakes synthesized through the LiF/HCl‐etching method,^[^ ^34^, ^46^ ^]^ whereas the latter contained positively charged polyelectrolyte polydiallyldimethylammonium chloride (PDDA) polymer. To construct multilayered films on the target substrate, the process of depositing a single‐monolayer MXene film was repeated, which involved four steps. First, the silica substrate with a negatively charged surface was immersed in the prepared polymer solution, resulting in the formation of a polymer‐coated substrate with a positively charged surface. Second, the polymer‐coated substrate was rinsed with a stream of deionized distilled water and then dried using N_2_. Third, the polymer‐coated substrate was submerged in the prepared MXene solution, allowing for the formation of an MXene monolayer on the upper surface driven by electrostatic forces. Finally, the MXene‐coated substrate was rinsed with a stream of deionized distilled water and then dried using N_2_. In Figure 1b we also provide a 3D profilometer image of a 2D layered MXene film coated on a silica substrate, which clearly shows the layered film structure. Unlike the cumbersome transfer processes used for coating other 2D materials such as graphene and TMDCs,^[^ ^47^, ^48^ ^]^ our method enabled transfer‐free and layer‐by‐layer coating of MXene films on dielectric substrates, together with high scalability and accurate control of the layer number or the film thickness. This coating method also shows high compatibility with integrated devices. Previously, we used a similar method to coat 2D layered GO films and successfully demonstrated many functional integrated photonic devices.^[^ ^16^, ^17^, ^30^, ^49^, ^50^, ^51^ ^]^ The self‐assembly method was employed to fabricate layered graphene and MoS_2_ films.^[^ ^52^, ^53^, ^54^ ^]^

Figure 1c shows a microscope image of monolayer MXene film coated on a silica substrate, which exhibits a high film uniformity. Figure 1d shows a picture of a 10‐layer MXene film coated on a ≈2 cm × 2 cm silica substrate, where a high degree of homogeneity was observed over the entire substrate, reflecting the capability of our MXene film coating method for coating in large areas. Figure 1e shows the Raman spectra of the MXene films with different layer numbers *N* = 5, 10, 15, 20, and 30, which were measured using a ≈514 nm pump laser. In our following discussion, the layer number refers to the number of monolayer MXene in a film. For all the samples, two narrow peaks at around ≈203 and ≈734 cm^−1^ were observed. As the layer number increased, the magnitude of the peaks also increased. These results are consistent with previous measurements for MXene films in refs. [55, 56], which verify the high quality of our prepared MXene films.

**Figure 2:**
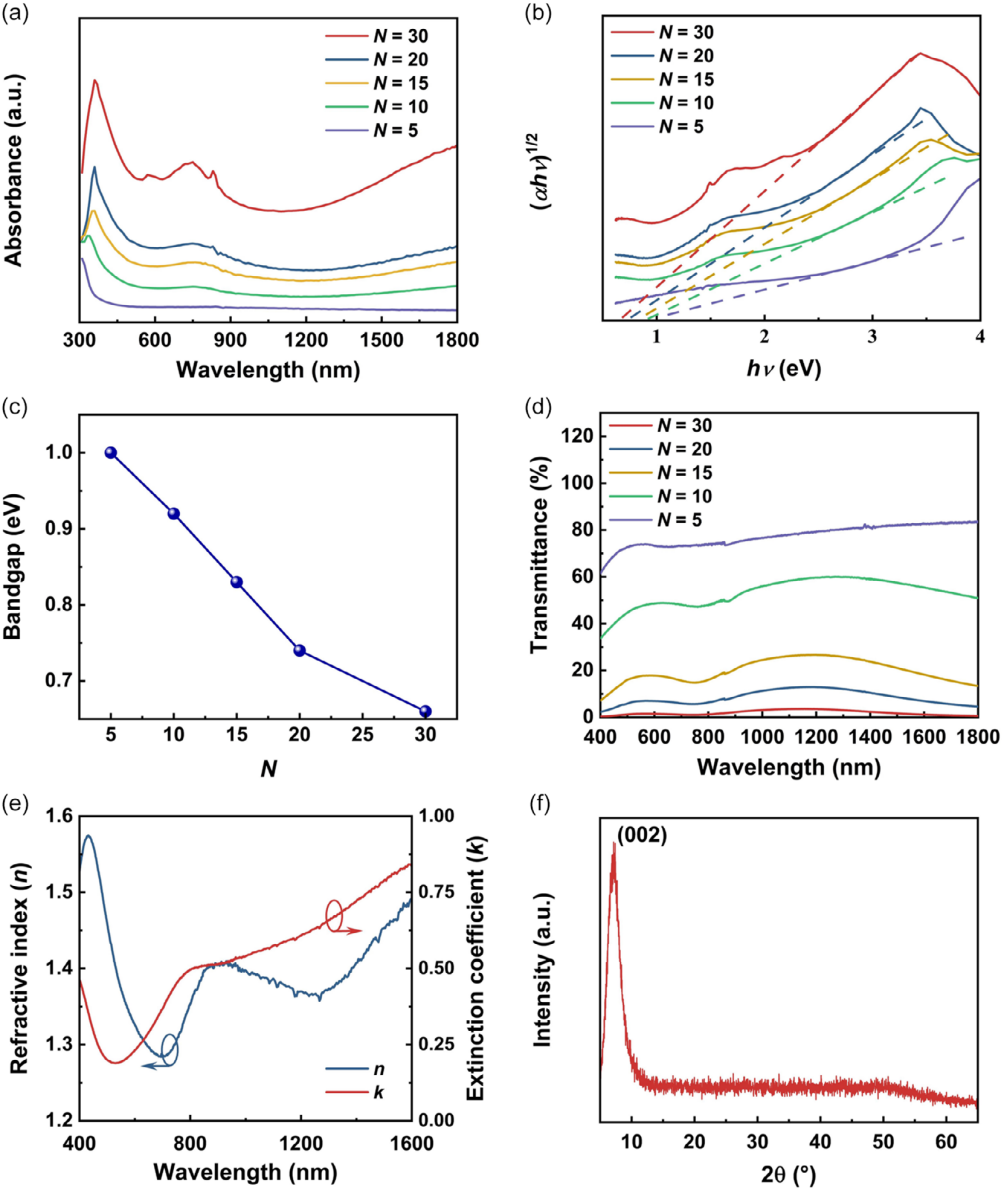
Characterization of the prepared MXene films. a) UV–vis absorption spectra for samples with different layer numbers *N*. b) The Tauc plot extracted from the absorption spectra in (a). c) Optical bandgap versus *N* extracted from (b). d) Linear transmittance spectra for samples with different layer numbers *N*. e) Measured in‐plane refractive index *n* and extinction coefficient *k* for the 5‐layer sample. f) XRD spectrum of a 5‐layer MXene film.

**Figure** **2**a shows the optical absorption spectra of the MXene films with different layer numbers *N*, which were characterized by ultraviolet–visible (UV–vis) spectrometry. The linear absorption spectrum when *N* = 5 decreased sharply at wavelengths <400 nm and exhibited a low absorption at wavelengths >600 nm. In contrast, when *N* > 5, the absorption spectra rose rapidly and then fell rapidly in the range of 300–500 nm. In addition, the linear absorption of the samples increased with the increase of layer number *N*.

The optical bandgap of the MXene film can be estimated from a Tauc plot of (*αhv*)^1/2^ versus *hv* using the Tauc formula,^[^ ^57^ ^]^ where *α* and *h* are the optical absorption coefficient and photon energy, respectively. Figure 2b shows the Tauc plot extracted from the linear absorption spectra in Figure 2a, and Figure 2c shows the optical bandgap versus layer number further derived from Figure 2b. As the layer number *N* increased from 5 to 30, the optical bandgap of the MXene films decreased from ≈1 to ≈0.66 eV. We also noted that the bandgaps of our prepared MXene films were slightly higher than the values reported in the previous literature.^[^ ^36^ ^]^ This was possibly due to the presence of titanium oxide on the surface of MXene.^[^ ^58^ ^]^ Furthermore, different fractions of the surface functional groups could also result in the variations in the bandgap.^[^ ^59^ ^]^

Figure 2d shows the transmittance spectra of the MXene films with different layer numbers *N* = 5, 10, 15, 20, and 30. The transmittance of the samples decreased with an increasing layer number. The 5‐layer sample had a transmittance >60% at wavelengths between 400 nm and 1800 nm, which was consistent with previous results in refs. [22, 60].

Figure 2e shows the in‐plane refractive index *n* and extinction coefficient *k* of the 5‐layer MXene film characterized by spectral ellipsometry. Since the out‐of‐plane response of the thin samples is much weaker, we could only measure the in‐plane *n* and *k* of the MXene film. The refractive index first increased sharply, reaching its peak at ≈500 nm. It then experienced a sharp decline, reaching a minimum at ≈700 nm, followed by a fluctuating rise. This shows an agreement with the trend of the UV–vis absorption spectra in Figure 2a and confirms the validity of our ellipsometry measurements. The trend for *k* is opposite to that of *n*, with *k* falling sharply and reaching a minimum at ≈600 nm and then increasing rapidly before slowing down at ≈800 nm. Our measured *k* values were lower than that in ref. [38], which can be attributed to the differences in the sample size and the surface functional groups of the MXene films. In addition to the 5‐layer sample, we conducted measurements on other samples with different layer numbers. The measured values for *n* were nearly identical to those obtained for the 5‐layer sample. The measured *k* values exhibited a minor rise as the layer number increased. For instance, at a wavelength of ≈800 nm, the measured *k* values for the samples including 5 and 30 layers were ≈0.49 and ≈0.53 respectively.

Figure 2f shows the X‐ray diffraction (XRD) spectrum of a 5‐layer MXene film. The (002) diffraction peak at 2*θ* ≈ 7°, which corresponds to the basal planes of the 2D titanium carbide layers, shows an agreement with the measured XRD spectra in refs. [61, 62] and validates our successful preparation of the MXene film.

## 3 *Z*‐Scan Measurements at ≈800 nm

**Figure 3:**
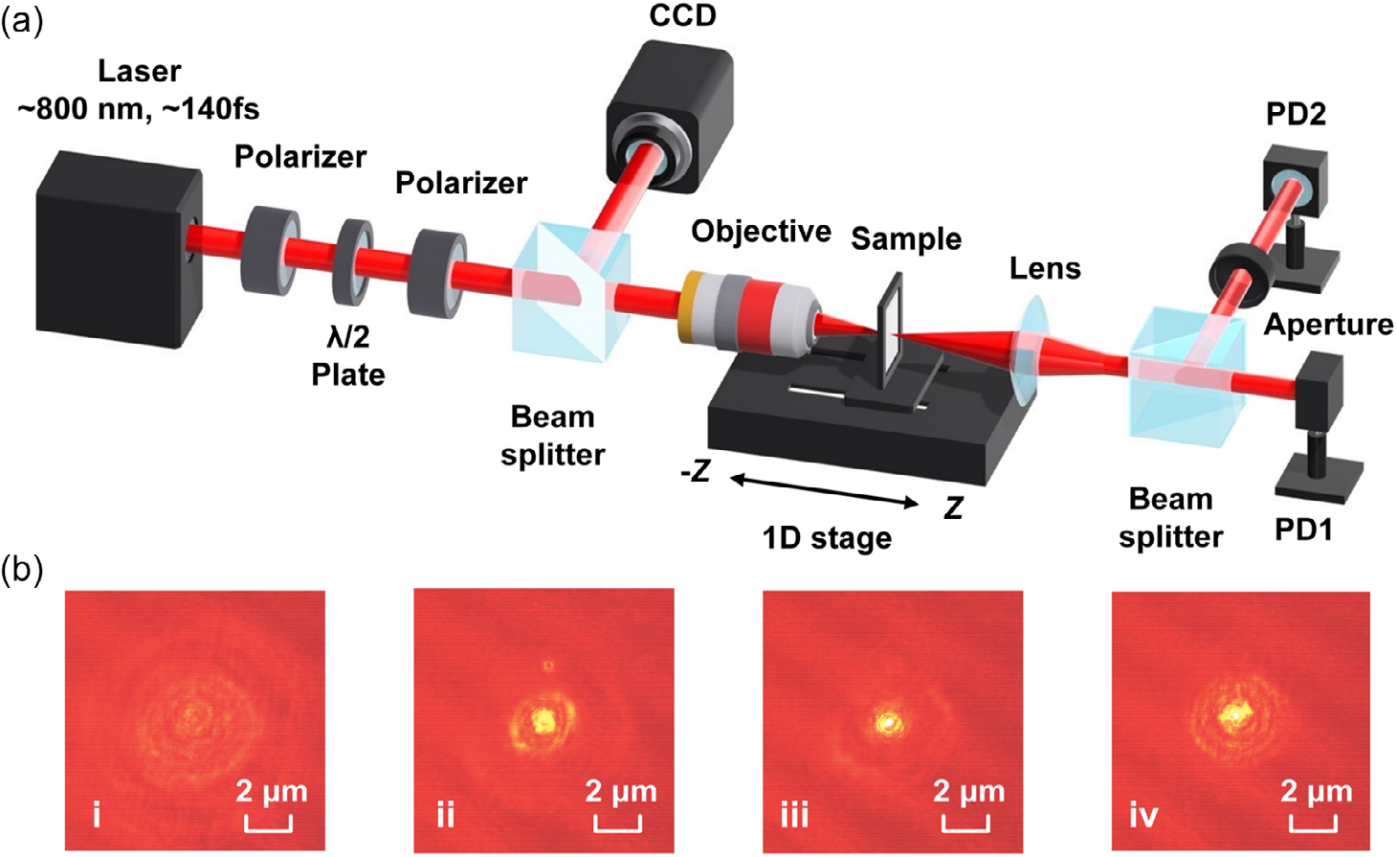
a) Schematic illustration of the *Z*‐scan experimental setup. CCD: charged‐coupled device. PD: photo detector. b) The focused laser beam on the sample at different positions along the *Z*‐axis, where *Z *= (i) −0.04 mm, (ii) −0.015 mm, (iii) 0 mm, and (iv) 0.015 mm, respectively.

The *Z*‐scan technique was employed to characterize the NOA of the MXene films that we prepared. **Figure** **3**a illustrates the experimental setup used for the *Z*‐scan measurements. The samples were excited using femtosecond optical pulses generated by an optical parametric oscillator, which had a center wavelength of ≈800 nm, a repetition frequency of ≈80 MHz, and a pulse duration of ≈140 fs. The utilization of a half‐wave plate in conjunction with a linear polarizer was implemented as a power attenuator to adjust the incident light power. The beam expansion system consisted of a ≈25 mm concave lens and two ≈150 mm convex lenses, which were utilized to expand the light beam. The expanded beam was then focused by an objective lens (10×, 0.25 NA), resulting in a focused spot size of ≈1.6 μm. The prepared sample to be measured was positioned at a right angle to the direction of the beam axis and subsequently moved along the *Z*‐axis using a highly precise 1D linear motorized stage. The alignment of the light beam to the target sample was achieved through a high‐definition charge‐coupled device (CCD) imaging system. Two PDs were utilized to measure the power of the transmitted light. Similar to our prior measurements on GO films,^[^ ^63^ ^]^ BP,^[^ ^20^ ^]^ BiOBr nanoflakes,^[^ ^64^ ^]^ PdSe_2_,^[^ ^65^ ^]^ and CH_3_NH_3_PbI_3_ perovskite nanosheets,^[^ ^66^ ^]^ the *Z*‐scan setup was calibrated to achieve a high accuracy before our *Z*‐scan measurements.

In the open‐aperture (OA) measurement, all transmitted light passing through the sample was collected by PD1 in Figure 3a, and the observed variation in the optical transmittance was induced by the NOA of the sample. To determine the nonlinear absorption coefficient (*β*) of the MXene film, the measured OA results were fit with^[^ ^64^, ^65^ ^]^

(1) $${\text{T}}_{\text{OA}}(\text{Z})\;≃\text{ 1–}\frac{1}{2\sqrt{2}}\frac{{\text{βI}}_{0}L_{\text{eff}}}{\text{1}+\text{Z}\text{/}{\text{Z}}_{0}}$$

where *T*_OA_(*Z*) is the normalized optical transmittance of the OA measurement, *I*_0_ is the irradiance intensity at the focus, *Z* and *Z*_0_ are the sample position relative to the focus and the Rayleigh length of the laser beam, respectively, and $L_{\text{eff}}=(1-e^{-\alpha_{0}L})/\alpha_{0}$ is the effective sample thickness, with *α*_0_ and *L* denoting the linear absorption coefficient and the sample thickness, respectively.

Figure 3b shows images of the focused laser beam on the sample at different positions along the *Z*‐axis, which were recorded by the CCD camera in Figure 3a. The incident laser intensity was ≈53.29 GW cm^−2^, and no visible damages or changes were observed in the sample as a result. A hazy outline of the beam was observed when the sample was initially out of focus, and the radius of the beam was dispersed throughout the boundaries of the image. As the sample approached the focal point at *Z* = 0 mm, the beam radius decreased, and the center became more brilliant. The beam became a brilliant speckle with a diameter of ≈1.6 μm when the sample was at the focus point. Subsequently, the beam spread as the sample moved away from the focal point.

**Figure 4:**
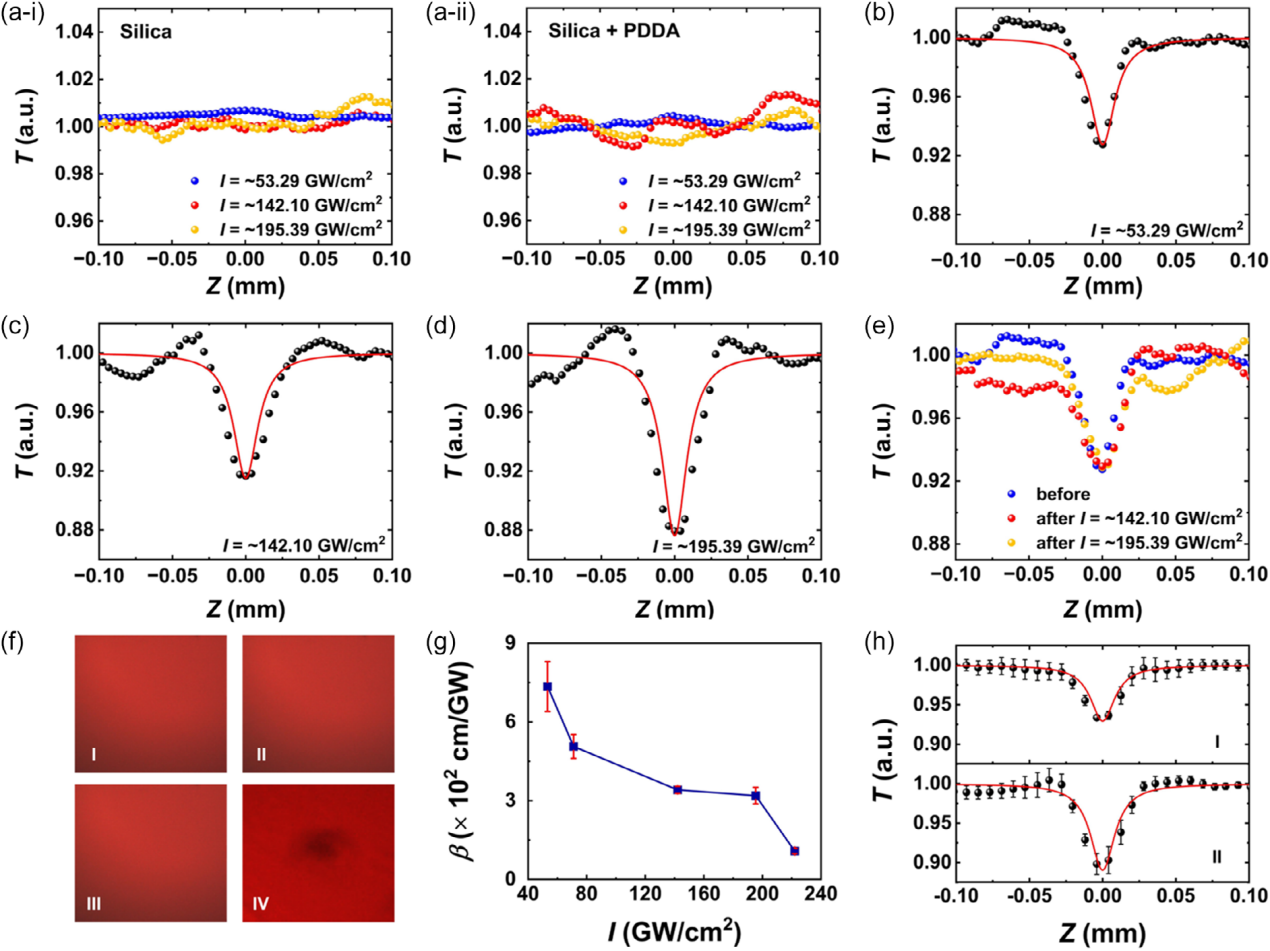
a) Measured OA results for (i) an uncoated silica substrate and (ii) a PDDA polymer‐coated silica substrate at different irradiance laser intensities of *I* = ≈53.29, ≈142.10, and ≈195.39 GW cm^−2^, respectively. *T*: normalized transmittance. b–d) OA *Z*‐scan results of a 5‐layer MXene film at *I* = ≈53.29, ≈142.10, and ≈195.39 GW cm^−2^, respectively. e) Measured OA results of a 5‐layer MXene film at *I* = ≈53.29 GW cm^−2^. The three curves were recorded before and after high‐power measurements at *I* = ≈142.10 and ≈195.39 GW cm^−2^. f) Images of the surface of a 5‐layer MXene film before and after the *Z*‐scan measurements. (I) The result before the *Z*‐scan measurements, and (II)–(IV) the results after the *Z*‐scan measurements at *I* = ≈53.29, ≈195.39, and ≈444.07 GW cm^−2^, respectively. g) Nonlinear absorption coefficient *β* of the MXene film versus *I*. The data points show the averaged values for measurements taken at five distinct locations on the sample, with the error bars indicating the variations among these measurements. h) OA results with averaged data points and error bars. (I) and (II) The results corresponding to *I *= ≈53.29 and ≈195.39 GW cm^−2^ in (g), respectively.

**Figure** **4**a shows the OA results for both an uncoated silica substrate and a PDDA‐polymer‐coated silica substrate, measured at incident laser intensities ranging from ≈53.29 to ≈195.39 GW cm^−2^. In both cases, no prominent peaks or notches were observed in the measured curves, indicating that neither the silica substrate nor the polymer caused any significant NOA.

Figure 4b–d shows the OA results for a 5‐layer MXene film measured at varying incident laser intensities ranging from ≈53.29 to ≈222.04 GW cm^−2^, respectively. In the OA curves, typical RSA, or optical limiting behavior was observed, with the transmission decreasing as the MXene sample approached the focal point. In addition, it was observed that the transmittance dip of the OA curve decreased as the incident laser intensity increased. In contrast, we did not observe any significant NOA for an uncoated silica substrate and a silica substrate only coated with PDDA polymer, indicating that the MXene film was responsible for the observed NOA. The modest deviation of experimental results from the standard symmetric OA curves can be attributed to the scattering from minor particles on the MXene samples as well as the irregularities and asymmetries in the input laser beam profile.

We employed three methods to detect damages to the MXene films. First, following the *Z*‐scan measurements at high laser intensities of ≈142.10 and ≈195.39 GW cm^−2^, we reduced the incident laser intensity to ≈53.29 GW cm^−2^ and repeated the measurements at the same location on the MXene film. Figure 4e shows the OA curves recorded before and after the *Z*‐scan measurements at high laser intensities. The three curves remain consistent except for minor differences, and the light transmittance at *Z* = 0 mm remains nearly constant at ≈0.93. These results provide evidence that there were no significant damages to the MXene films during the *Z*‐scan measurements. Second, we used a CCD camera (as shown in Figure 3) to capture images of the MXene film surface before and after conducting the *Z*‐scan measurements at different laser intensities. As shown in the Figure 4f, there were no obvious differences in the film surface before and after our *Z*‐scan measurements at laser intensities of ≈53.29 and ≈195.39 GW cm^−2^. In contrast, upon further increasing the laser intensities to ≈444.07 GW cm^−2^, the film surface exposed to the laser beam exhibited noticeable darkening. These results further verify that the MXene films did not suffer from significant damages during our *Z*‐scan measurements with a maximum laser intensity of ≈195.39 GW cm^−2^. Finally, as the laser power increased beyond 400 GW cm^−2^, we observed a sudden rise in the measured SA peak in the OA curve. After this, the SA peak of the OA curve no longer broadened by further increasing the laser power. This phenomenon observed in our measurements can serve as another indicator of film damages, and this is also confirmed by observing a damaged film surface similar to that in Figure 4f.

By fitting the measured OA results with Equation (1), we derived the nonlinear absorption coefficient *β* of the MXene films. Figure 4g shows the fit values of *β* as a function of the irradiance laser intensity *I*. Five distinct locations on the sample were measured, and the data points represent their average values, with the error bars indicating the variations among these measurements. To better illustrate the variation among different measurements, the OA curves with averaged data points and corresponding error bars at *I* = ≈53.29 and ≈195.39 GW cm^−2^ are shown in Figure 4h. A large *β* value of ≈7.13 × 10^2^ cm GW^−1^ is achieved at *I* = ≈53.29 GW cm^−2^. It is also observed that absorption coefficient decreases as laser intensity increases. A similar phenomenon was previously reported in ref. [22], which could be attributed to the alterations in the surface functional groups of the MXene films induced by the increased laser intensity.

**Figure 5:**
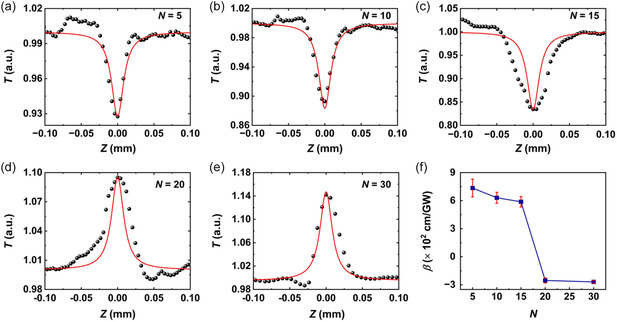
a–e) Measured (data points) and fit (solid curves) OA results for MXene films with different layer numbers *N* = 5, 10, 15, 20, and 30. *T*: normalized transmittance. f) Fit the nonlinear absorption coefficient *β* versus layer number *N*.

**Figure** **5**a–e depicts the OA results for MXene films with different layer numbers *N* = 5, 10, 15, 20, and 30. For comparison, all the samples had the same irradiance laser intensity of ≈53.29 GW cm^−2^. For the measured OA curves in Figure 5, similar phenomena as discussed in Figure 4e,f were observed to verify the absence of significant damages to all the MXene films at an incident laser intensity of ≈53.29 GW cm^−2^. The fit *β* as a function of *N* is shown in Figure 5f. Similar to that in Figure 4g, we also measured five distinct locations for each sample. As *N* increases from 5 to 30, the average value of the fit *β* changes from ≈7.13 × 10^2^ cm GW^−1^ to ≈−2.69 × 10^2^ cm GW^−1^. It is interesting to observe that the NOA behavior of the MXene film exhibits significant layer dependence. For *N* ≤ 15, the film displays typical RSA behavior, whereas SA behavior is observed for *N* ≥ 20.

We also characterized the nonlinear refractive index *n*_2_ of the MXene films using the closed aperture measurement. A small aperture was positioned before PD2 in Figure 3 to collect only a portion of the on‐axis transmitted light beam. However, we did not observe any significant *z*‐scan curves, indicating that the *n*_2_ is not high. Our observation here is consistent with the result in ref. [22], where the measured *n*_2_ value of MXene was ≈−10^−20^ m^2^ W^−1^ and was two orders of magnitude lower than that of silicon.^[^ ^1^ ^]^

## 4 On‐Chip Integration and Characterization of NOA at ≈1550 nm

**Figure 6:**
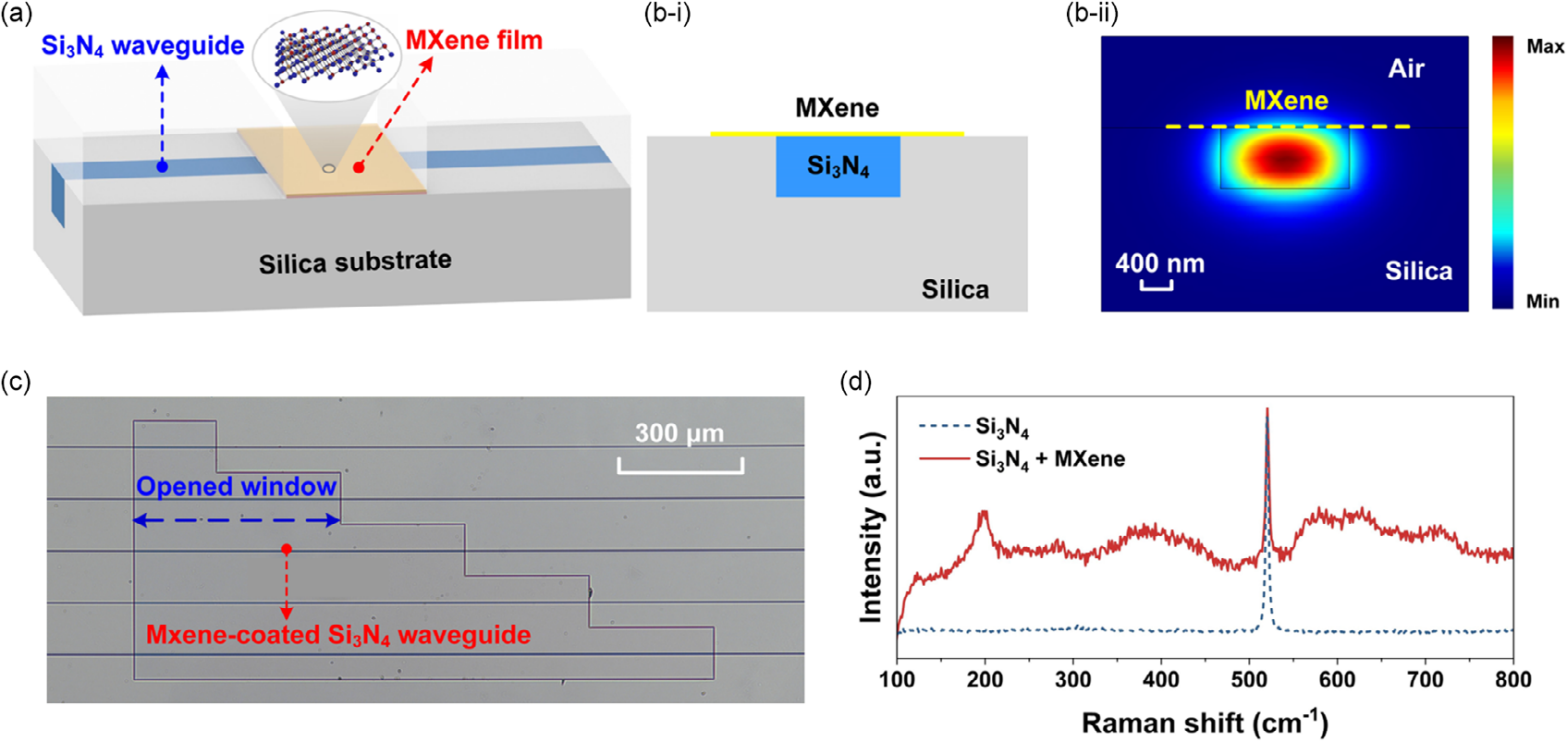
a) Schematic illustration of a Si_3_N_4_ waveguide integrated with a MXene film. b–i) Schematic illustration of cross section and b–ii) corresponding TE mode profile of a hybrid waveguide with a 5‐layer MXene film. c) Microscope image of Si_3_N_4_ integrated chip uniformly coated with a 5‐layer MXene film. d) Measured Raman spectra of the Si_3_N_4_ chip in (c) before and after coating the MXene film.

In this section, we characterize the NOA of 2D layered MXene films by integrating them onto Si_3_N_4_ waveguides. **Figure** **6**a shows the schematic of a Si_3_N_4_ waveguide coated with a MXene film. Here, we choose Si_3_N_4_ waveguides because Si_3_N_4_ has a large bandgap of ≈5 eV^[^ ^1^ ^]^ that yields negligible NOA at near‐infrared wavelengths. In our fabrication, the Si_3_N_4_ waveguides were fabricated using CMOS‐compatible and crack‐free processes, as reported previously.^[^ ^67^, ^68^ ^]^ ≈2.3 μm‐thick silica layer was deposited onto the Si_3_N_4_ waveguides as an upper cladding, followed by a window opening on it via the lithography and dry etching processes to allow the deposition of MXene films onto the waveguide top surface. The coating of the 2D MXene films was realized using the solution‐based coating method illustrated in Figure 1b, which enabled transfer‐free and layer‐by‐layer film coating onto the Si_3_N_4_ waveguides.

Figure 6b–i shows the schematic cross section of the hybrid waveguide with a 5‐layer MXene film. The corresponding transverse electric (TE) mode profile is shown in Figure 6b–ii. The width and height of the Si_3_N_4_ waveguide were 1.60 and 0.72 μm, respectively. The waveguide evanescent field can excite NOA in the coated MXene film, which can be characterized by measuring the output from the hybrid waveguide. We selected TE polarization for our subsequent measurements since it supports in‐plane interaction between the MXene film and the waveguide evanescent field, which is much stronger than the out‐of‐plane interaction given the significant anisotropy of 2D materials.^[^ ^6^, ^50^ ^]^

Figure 6c shows a microscopic image of the window opening area on a Si_3_N_4_ chip that was uniformly coated with a 5‐layer MXene film. The coated MXene film exhibits good morphology as well as a high uniformity without any noticeable wrinkling or stretching. Figure 6d shows the measured Raman spectra of the Si_3_N_4_ chip in Figure 6c before and after coating the MXene film. The presence of the representative peaks around ≈203 and ≈734 cm^−1^ in the MXene‐coated chip is consistent with those in Figure 1e and confirms the successful on‐chip integration of the MXene film.

**Figure 7:**
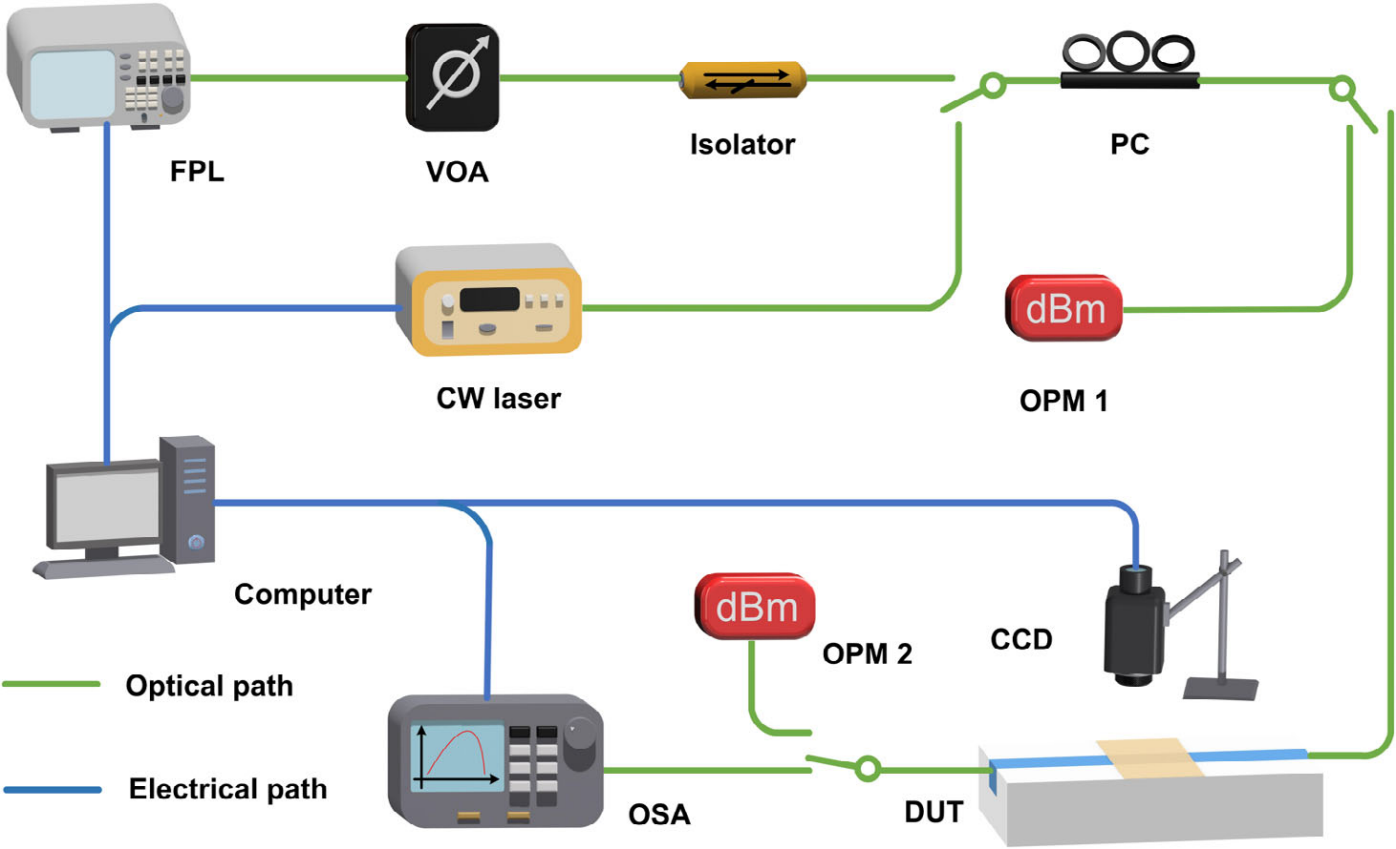
Experimental setup for measuring NOA of MXene‐coated Si_3_N_4_ waveguides. CW laser. FPL: fiber pulsed laser. VOA: variable optical attenuator. PC: polarization controller. OPM: optical power meter. DUT: device under test. CCD: charged‐coupled device. OSA: optical spectrum analyzer.

**Figure** **7** shows the experimental setup used for measuring the NOA of the MXene‐coated Si_3_N_4_ waveguides. Two laser sources were employed, including a tunable continuous‐wave (CW) laser and a fiber pulsed laser (FPL) capable of generating nearly Fourier‐transform‐limited femtosecond optical pulses centered at ≈1550 nm. An optical isolator was used to prevent the reflected light from damaging the laser source, and a variable optical attenuator (VOA) was employed to tune the power of input light. A polarization controller (PC) was inserted before the device under test (DUT) to adjust the input light to TE polarization. Inverse‐taper couplers were fabricated at both ends of the Si_3_N_4_ waveguides, which were butt coupled to lensed optical fibers to achieve light coupling into and out of the DUT. The fiber‐to‐chip coupling loss was ≈4 dB per facet. To characterize the NOA in the DUT, the power of the light before and after passing was measured by two optical power meters, that is, OPM 1 and OPM 2.

**Figure 8:**
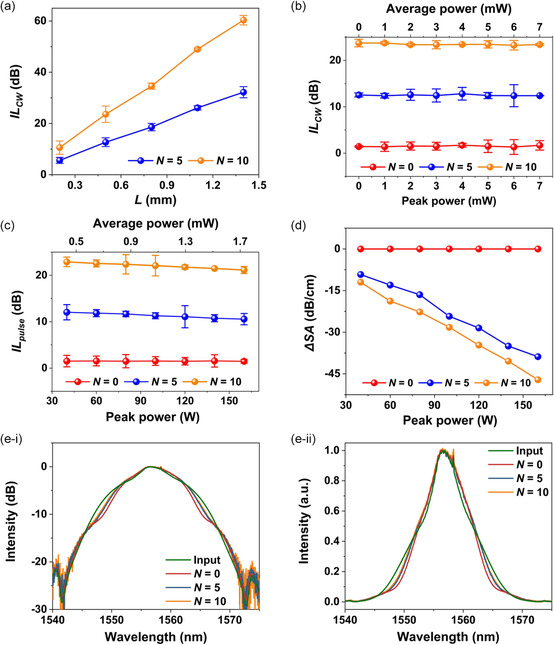
a) Measured insertion loss (*IL*_CW_) versus MXene film length (*L*) for the hybrid waveguides with films including 5 and 10 layers of MXene (*N* = 5, 10). The input power of the CW light remained constant at ≈0 dBm. b) Measured *IL*_CW_ versus input power of CW light for the uncoated (*N* = 0) and hybrid waveguides. c) Measured insertion loss (*IL*_pulse_) versus peak/average power of input optical pulses for the uncoated and hybrid waveguides. d) Excess propagation loss induced by the SA (Δ*SA*) versus peak power of input optical pulses extracted from (c). In (a)−(d), the data points depict the average of measurements on three samples and the error bars illustrate the variations among the different samples. e) Normalized spectra of optical pulses before and after propagation through uncoated and hybrid waveguides. (i) and (ii) show the same results plotted in a logarithmic scale and a linear scale, respectively. The peak power of the input femtosecond optical pulses was ≈160 W.

We first measured the linear loss of the 2D MXene films. **Figure** **8**a shows the insertion loss (*IL*_CW_) versus MXene film length (*L*) for the hybrid waveguides with films including 5 and 10 layers of MXene, which was measured using a CW light at a fixed wavelength of ≈1550 nm. The total length of our fabricated Si_3_N_4_ waveguides was ≈20 mm and the length of the opened window (i.e., the MXene film length *L*) varied from ≈0.2 to ≈1.4 mm. The input power of the CW light remained constant at ≈0 dBm. Unless otherwise specified, the input power of CW light or optical pulses in our discussion refers to the power coupled into the waveguide after excluding the fiber‐to‐chip coupling loss. For both hybrid waveguides, the *IL*_CW_ increases with *L*. The extracted excess propagation losses induced by the MXene films were ≈20.13 and ≈39.27 dB mm^−1^ for the devices with 5 and 10 layers of MXene, respectively. These correspond to an average excess propagation loss of ≈40 dB cm^−1^ for each MXene layer. This value is higher than the excess propagation loss induced by monolayer GO (≈3 dB cm^−1^^[^ ^32^, ^68^ ^]^), but lower than that induced by monolayer graphene (≈500 dB cm^−1^^[^ ^69^ ^]^) coated on Si_3_N_4_ waveguides.

Figure 8b shows the measured *IL*_CW_ versus input CW power for the uncoated Si_3_N_4_ waveguide (*N* = 0) and the hybrid waveguides with 5 and 10 layers of MXene (*N* = 5, 10). For all the three devices, no significant changes in the *IL*_CW_ were observed for input power below ≈7 mW. This reflects a negligible power‐dependent loss due to photothermal changes (which are sensitive to the average light power^[^ ^30^, ^32^ ^]^) in the MXene films within this power range.

Following the measurements of linear loss, we measured the nonlinear loss of the MXene films using femtosecond optical pulses generated by the FPL. Unlike CW light, which has a peak power equal to its average power, optical pulses exhibit peak powers significantly higher than their average powers. In our experiments, the femtosecond optical pulses had a repetition rate of ≈60 MHz and a pulse duration of ≈180 fs. The average input power varied between ≈0.4 and ≈1.7 mW, corresponding to a peak power range of ≈40–≈160 W. The maximum average power was less than ≈7 mW. According to the results in Figure 8b, there was no significant power‐dependent loss induced by photothermal changes in this power range.

Figure 8c shows the measured insertion loss (*IL*_pulse_) versus the input power of femtosecond optical pulses. Here we show the results for three devices, including the uncoated Si_3_N_4_ waveguide (*N* = 0) and the hybrid waveguides with 5 and 10 layers of MXene (*N* = 5, 10). It can be seen that the insertion loss of the two hybrid waveguides decreases with increasing pulse peak power, with the 10‐layer device showing a more significant decrease than the 5‐layer device. In contrast, the insertion loss of the uncoated Si_3_N_4_ waveguide remains nearly constant. These results reflect that there was *SA* induced by the MXene films in the hybrid waveguides.

Figure 8d shows the SA‐induced excess propagation loss (Δ*SA*, after excluding the linear propagation loss) versus pulse peak power, which was extracted from the results in Figure 8b,c. The negative values of Δ*SA* indicate that the loss decreases as the peak power increases. Based on the Δ*SA* in Figure 8d, we calculate the effective nonlinear absorption coefficient (*β*_eff_) of the hybrid waveguide using the equation below.^[^ ^70^ ^]^

(2) $$\alpha_{\text{SA}}\;≃\;\frac{\beta_{\text{eff}}\;\text{P}\text{ (}\text{z}\text{)}}{A_{\text{eff}}}$$

where *α*_SA_ is the loss factor (in units of m^−1^) corresponding to Δ*SA* (in units of dB cm^−1^), *A*_eff_ is the effective mode area, and *P* (*z*) is the pulse peak power which attenuates along the waveguide in the *z*‐axis. The calculated *β*_eff_ values were ≈−0.60 and ≈−1.17 GW cm^−1^ for the 5‐layer and 10‐layer MXene devices, respectively. The *β* values of the MXene films were further extracted from these *β*_eff_ values based on^[^ ^31^ ^]^

(3) $$\beta_{\text{eff}}=\frac{\iint_{D}n^{\text{2 }}{(x,\;y)\beta \;(x,\;y)S}_{z}^{2}dxdy}{{\left[\iint_{D}{n(x,\;y)S}_{z}dxdy\right]}^{2}}$$

where *D* is the integral of the optical fields over different material regions, *S*_*z*_ is the time‐averaged Poynting vector, and *n* (*x*, *y*) and *β* (*x*, *y*) are the linear refractive index and nonlinear absorption coefficient profiles over the waveguide cross section. In our calculation, the values of *n* for Si_3_N_4_ and MXene were ≈1.99 and ≈1.46 at ≈1550 nm, respectively. The former one was obtained from our previous measurements on similar Si_3_N_4_ devices,^[^ ^32^ ^]^ and the latter one was obtained from our ellipsometry measurements in Figure 2e. We also assumed that *β* = 0 for Si_3_N_4_ at ≈1550 nm given its large optical bandgap. For the films including 5 and 10 layers of MXene, the obtained *β* values were ≈−1.76 × 10^2^ and ≈−1.81 × 10^2^ GW cm^−1^, respectively. It is interesting to note that these results at ≈1550 nm have a sign opposite to the corresponding results at ≈800 nm (i.e., ≈7.34 × 10^2^ and ≈6.31 × 10^2^ GW cm^−1^ for the film including 5 and 10 layers of MXene, respectively). This reflects the fact that the NOA behavior of the MXene film exhibits significant wavelength dependence, which transitions from RSA to SA as the wavelength changes from ≈800 to ≈1550 nm.

We also characterized the Kerr nonlinearity of the MXene films using the optical spectrum analyzer (OSA) in Figure 7 to measure the spectral broadening of the output from the hybrid waveguides. Figure 8e shows the normalized spectra of femtosecond optical pulses before and after propagation through the uncoated and hybrid waveguides. To better identify the spectral broadening, in (i) and (ii) the same results were plotted in a logarithmic scale and a linear scale, respectively. The peak power of the input femtosecond optical pulses was kept the same at ≈160 W. As compared with the input pulse spectrum, the output spectrum after propagation through the uncoated Si_3_N_4_ waveguide exhibited measurable spectral broadening induced by the Kerr nonlinearity of Si_3_N_4_. On the other hand, the outputs from the hybrid waveguides with 5 and 10 layers of MXene did not show obvious spectral broadening as compared to the output from the uncoated waveguide. This reflects that the *n*_2_ of the MXene films is not high, which is consistent with our *Z*‐scan measurement results at ≈800 nm.

To quantitatively characterize the *n*_2_ of the MXene films, we calculated the theoretical nonlinear spectral broadening using a split‐step Fourier method to solve the nonlinear Schrodinger equation as follows.^[^ ^71^ ^]^

(4) $$\frac{\partial A}{\partial \text{z}}\text{= –}\frac{i{\text{β}}_{2}}{2}\frac{\partial^{2}A}{\partial {\text{t}}^{2}}\text{+ i}\text{γ}|A{|}^{2}A–\frac{1}{2}\;\text{α}A$$

where *i *= $\sqrt{1}$, *A* (*z*, *t*) is the slowly varying temporal pulse envelope along the propagation direction *z*, *β*_2_ is the second‐order dispersion coefficient, and *γ* is the waveguide nonlinear parameter. The total loss factor *α* includes both the linear propagation loss and the SA‐induced excess propagation loss in Figure 8d. In Equation (4), we retain only the second‐order dispersion *β*_2_ given that the dispersion length (>1 m) is much longer than the physical length of the waveguides (≈20 mm). In our calculations, the MXene‐coated Si_3_N_4_ waveguides were divided into uncoated (with silica cladding) and MXene‐coated segments. For each segment, Equation (4) was numerically solved, and the output *A* from the previous segment was set as the input for the following one.

Using theoretical calculations based on Equation (4) to fit the results Figure 8e, we obtained the fit *γ* values for the uncoated and hybrid waveguides. The fit *γ* for the uncoated Si_3_N_4_ waveguide is ≈1.5 W^−1^ m^−1^, which is consistent with previously reported values.^[^ ^32^ ^]^ On the other hand, the fit *γ* values for the hybrid waveguides with 5 and 10 layers of MXene are ≈1.8 and ≈2.1 W^−1^ m^−1^, respectively, which are ≈1.3 and ≈1.4 times that of the uncoated Si_3_N_4_ waveguide. The accuracy of fitting *γ* is compromised by the nonideal shape of the optical pulses’ spectra, especially when there is minimal nonlinear spectral broadening in Figure 8e. Therefore, there could be discrepancies for the fit *γ* values. Even if these discrepancies can be excluded, the maximum *γ* values will not exceed 2.4 and 3.0 W^−1^ m^−1^ for the 5‐ and 10‐layer devices, respectively.

Based on the fit *γ* values of the hybrid waveguides, we further extracted the *n*_2_ of the layered MXene films using^[^ ^31^ ^]^

(5) $$\gamma \text{ = }\frac{\text{2}\pi }{\lambda_{c}}\frac{\iint_{D}n^{\text{2 }}{(x,\;y)n}_{2}{(x,\;y)S}_{z}^{2}dxdy}{{\left[\iint_{D}{n\;(x,\;y)S}_{z}dxdy\right]}^{2}}$$

where *λ*_c_ is the pulse central wavelength and *n*_2_ (*x*, *y*) is the Kerr coefficient of the different material regions. The values of *n*_2_ for silica and Si_3_N_4_ used in our calculation were 2.6 × 10^−20^ m^2^ W^−1^^[^ ^1^ ^]^ and 2.5 × 10^−19^ m^2^ W^−1^, respectively, with the latter obtained by fitting the experimental results for the uncoated Si_3_N_4_ waveguide. The extracted *n*_2_ values for the film including 5 and 10 layers of MXene are ≈2.1 × 10^−16^ m^2^ W^−1^ and ≈2.8 × 10^−16^ m^2^ W^−1^, respectively. Considering the discrepancies in the fit *γ* values, the maximum *n*_2_ values for these MXene films should be lower than ≈4 × 10^−16^ m^2^ W^−1^. This magnitude is much lower than the magnitudes for the *n*_2_ of graphene (≈−1 × 10^−13^ m^2^ W^−1^^[^ ^72^ ^]^) and GO (≈1.28 × 10^−14^ m^2^ W^−1^^[^ ^32^ ^]^), which further confirms the relatively low Kerr nonlinearity for the MXene films.

## 5 Discussion

According to the UV–vis spectra in Figure 2a, the bandgap of the 5‐layer MXene film is estimated to be ≈1.0 eV, which is lower than the single‐photon energy of the incident light at ≈800 nm, that is, ≈1.55 eV. As a result, the NOA behavior when *N* = 5 should be SA, while our *Z*‐scan measurement result in Figure 5a shows a positive *β* that corresponds to the RSA. In principle, the RSA can be induced by various nonlinear optical effects such as nonlinear light scattering (NLS), two‐photon absorption (TPA), multiphoton absorption, excited‐state absorption (ESA), and multiphoton absorption. These effects could coexist in practical MXene films, thus resulting in complex and wavelength‐dependent NOA behavior. Since the NLS effect typically dominates in dispersion and solution‐based materials with laser‐induced microbubbles,^[^ ^65^ ^]^ it should not be a significant factor for our prepared MXene films. Considering the relatively low efficiency of multiphoton absorption, TPA and ESA are likely responsible for the observed RSA in our case.

**Figure 9:**
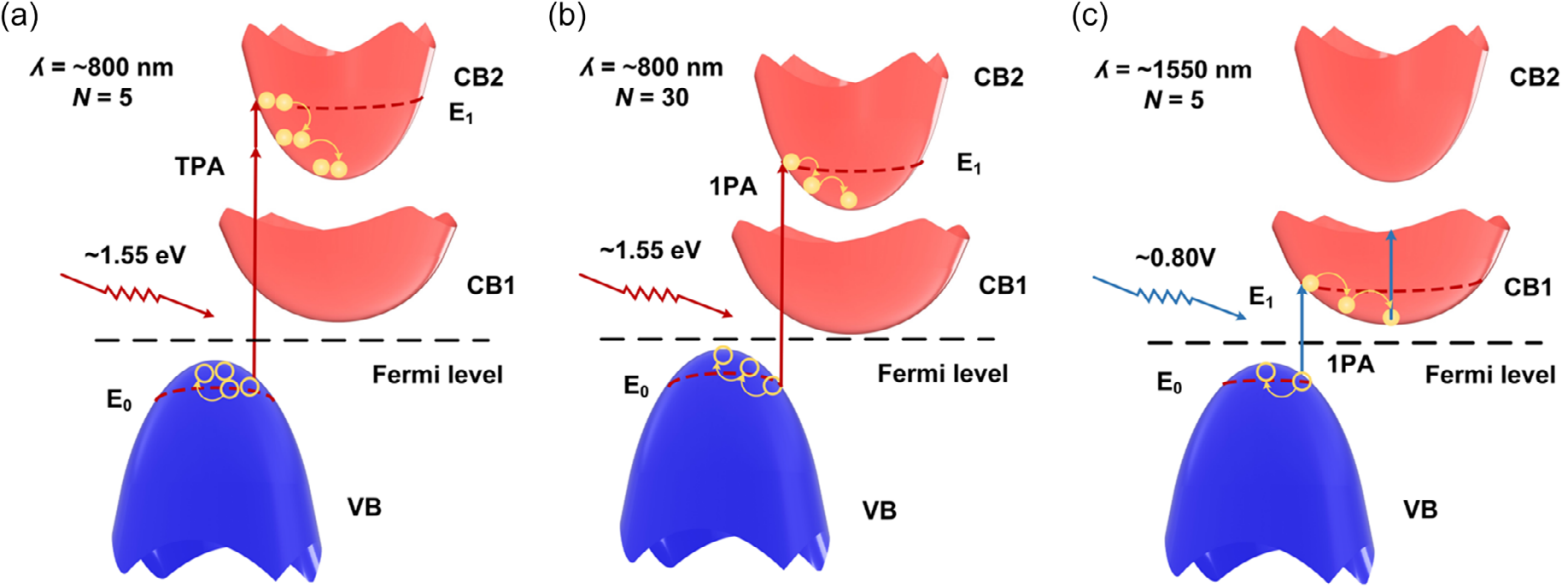
Schematic illustration of bandgap structures for a) 5‐layer MXene films at ≈800 nm, b) 30‐layer MXene films at ≈800 nm, and c) 5‐layer MXene films at ≈1550 nm. 1PA: one‐photon absorption. TPA: two‐photon absorption. VB: valence band. CB1: the first excited state in the conduction band. CB2: the second excited state in the conduction band.

Given the challenges in probing the specific mechanisms within MXene films through *Z*‐scan measurements, we use the schematic illustration of bandgap structures depicted in **Figure** **9** to discuss possible reasons for the layer‐dependent and wavelength‐dependent NOA observed in our experiments. In the bandgap structures, there are two excited states in the conduction band, CB1 and CB2. For MXene films with smaller thicknesses (i.e., *N* = 5), upon excitation by ≈800 nm (i.e., ≈1.55 eV) femtosecond optical pulses, electrons are first excited to CB1, followed by the continuous excitation of extra electrons to CB2 due to ESA, as illustrated in Figure 9a. This could result in the RSA behavior for the film at ≈800 nm.

As the film thickness increases (i.e., *N* ≥ 20), the NOA observed in Figure 5d,e changes to SA, which can be induced by thickness‐dependent fluctuations in the bandgap. As illustrated in Figure 9b, the bandgap of MXene decreases with the increase of layer number. This is also evidenced by the results in Figure 2b,c, where a smaller bandgap of ≈0.66 eV is for the 30‐layer sample. As the bandgap decreases, the ≈1.55 eV photons can directly excite electrons to CB2 through one‐photon absorption (1PA). As a result, 1PA becomes dominant among the NOA effects, leading to an SA behavior for thicker MXene films at ≈800 nm.

As the wavelength varied from ≈800 to ≈1550 nm, the NOA behavior of the 5‐layer film changes from RSA to SA. This can possibly be attributed to more efficient excitation of electrons to CB1 for a lower photon energy of ≈0.8 eV. As shown in Figure 9c, electrons at the top of the valence band (VB) first absorb the ≈0.8 eV photons and transit to CB1. After this, the excited electrons quickly relax to the bottom of CB1. Similar transition from RSA to SA as the wavelength increases was also observed for graphene–TiO_2_,^[^ ^73^ ^]^ selenium‐doped BP,^[^ ^74^ ^]^ and franckeite.^[^ ^75^ ^]^

Besides, there are other possible explanations for the layer‐dependent NOA of the MXene films at ≈800 nm. First, both TPA and 1PA could exist in the MXene films. For films with smaller thicknesses (i.e., *N* = 5), TPA prevails due to its self‐sufficiency, as opposed to 1PA, which relies on phonon assistance for its occurrence. As the bandgap decreases with increasing film thickness, the need for phonon assistance in 1PA diminishes due to a better alignment between the bandgap related to single‐photon energy and the excited photon energy. This alignment facilitates a more accessible excitation through 1PA. As a result, 1PA becomes dominant, leading to the SA behavior in thicker films. Similar behaviors were also observed for 2D WS_2_, MoS_2_, Bi_2_S_3_, and PtSe_2_ films with different thicknesses.^[^ ^76^, ^77^ ^]^ In addition, a previous study has suggested a relationship between TPA and the bandgap shape of MXene,^[^ ^23^ ^]^ which can also explain the layer‐dependent behavior of our MXene films. Specifically, the bandgap associated with single‐photon energy could appear at the steeper edges of the cone structure. In contrast, at twice the photon energy, the bandgap exhibits a flatter shape, which makes TPA more favorable compared to 1PA for the films with smaller thicknesses. As the bandgap decreases with increasing film thickness, the bandgap associated with single‐photon energy could also appear at the flatter edges of the cone structure. This results in the prevalence of 1PA over TPA and hence the SA behavior for thicker films.

The carrier interaction between adjacent MXene layers is another interesting topic. Given the fact that in our films fabricated based on self‐assembly the adjacent MXene layers were separated by a dielectric polymer layer, the carrier interaction between adjacent MXene layers should not be significant. However, the results in Figure 5 indicate that the layered MXene films exhibit notable changes in their NOA properties (from RSA to SA) as the layer number increases. Such layer number dependence was also observed for the measured bandgap in Figure 2c. The dependence of the material properties on the layer number implies that there are interlayer interactions, which in turn modify the material bandgap. Although currently we lack suitable instruments to precisely characterize the interlayer interactions in the MXene films, we believe this deserves further investigation and could be the subject of our future work.

In **Table** **1**, we compare our measured nonlinear absorption coefficient *β* of Ti_3_C_2_T_*x*_ films with the reported values of 2D MXenes, including not only Ti_3_C_2_T_*x*_, but also others such as Nb_2_C, Ta_2_C, and Ti_3_CN NSs. For a thin film including five layers of MXene, we obtained a positive *β* on the order of 10^2^ cm GW^−1^ at ≈800 nm. This value is more than four orders of magnitude larger than the previously reported value for Ti_3_C_2_T_*x*_ at the same wavelength.^[^ ^23^ ^]^ On the other hand, a negative *β* on the order of −10^2^ cm GW^−1^ was obtained for a film including 30 layers of MXene at ≈800 nm. The absolute magnitude is much greater than that of MXenes consisting of other early transition metal elements such as Ti, Nb, and Ta.^[^ ^22^, ^78^, ^79^, ^80^ ^]^ At ≈1550 nm, we obtained a negative *β* with an absolute magnitude on the order of 10^2^ cm GW^−1^, which is about three orders of magnitude larger than those of Ta_2_C and Ti_3_C_2_T_*x*_ reported in other studies.^[^ ^22^, ^79^ ^]^

**Table 1:** Comparison of nonlinear absorption coefficient *β* for 2D MXenes.

Material	Laser parameter	Film thickness	*β* [cm GW^−1^]	References
Nb_2_C	≈800 nm, ≈35 fs, ≈2 kHz	few‐layer	≈−8 × 10^−2^	[78]
Nb_2_C	≈1550 nm, ≈35 fs, ≈2 kHz	few‐layer	≈0.30	[78]
Ta_2_C	≈800 nm, ≈100 fs, ≈1 kHz	few‐layer	≈−0.42	[79]
Ta_2_C	≈1550 nm, ≈100 fs, ≈1 kHz	few‐layer	≈−0.13	[79]
Ti_3_CN NSs	≈800 nm, ≈95 ± 10 fs, ≈1 kHz	few‐layer	≈−5.4 × 10^−2^	[80]
Ti_3_CN NSs	≈1550 nm, ≈95 ± 10 fs, ≈1 kHz	few‐layer	≈0.31	[80]
Ti_3_C_2_T_*x*_	≈800 nm, ≈95 ± 10 fs, ≈1 kHz	few‐layer	≈−0.297	[22]
Ti_3_C_2_T_*x*_	≈1550 nm, ≈95 ± 10 fs, ≈1 kHz	few‐layer	≈−0.358	[22]
Ti_3_C_2_T_*x*_	≈515 nm, ≈35 fs, ≈1 kHz	few‐layer	≈1.53 × 10^2^	[24]
Ti_3_C_2_T_*x*_	≈515 nm, ≈35 fs, ≈1 kHz	few‐layer	≈−1.02 × 10^3^	[24]
Ti_3_C_2_T_*x*_	≈800 nm, ≈100 fs, ≈100 kHz	one‐layer	≈1.20 × 10^−2^	[23]
Ti_3_C_2_T_*x*_	≈800 nm, ≈140 fs, ≈80 MHz	5 layers	≈7.13 × 10^2^	This work
Ti_3_C_2_T_*x*_	≈800 nm, ≈140 fs, ≈80 MHz	30 layers	≈−2.69 × 10^2^	This work
Ti_3_C_2_T_*x*_	≈1550 nm, ≈140 fs, ≈80 MHz	5 layers	≈−1.73 × 10^2^	This work

At high repetition rates, photothermal effects arising from repeated pulses become increasingly significant, which may lead to potential material property changes and even damages.^[^ ^81^, ^82^ ^]^ In our case, although the repetition rate of our laser source is higher than those of other works in Table 1, we only observed significant damages to the MXene films at very high incident laser intensities. In previous reports,^[^ ^83^, ^84^ ^]^ it was found that the resultant temperature gradient induced by high repetition rates could introduce inaccuracies in the measured *n*_2_ using the *Z*‐scan method. However, in our scenario, as the change in the measured *n*_2_ was not significant, the impact of the repetition rate should not be significant either. In addition, it is possible to differentiate thermal contributions to nonlinear refraction using temporal discrimination techniques and using a reference sample.^[^ ^83^ ^]^ Additionally, optimizing the chopping frequency and modulation duty cycle can effectively mitigate the photothermal effects.^[^ ^85^ ^]^

## 6 Conclusion

In summary, we prepare layered MXene films via a solution‐based method that yields transfer‐free and layer‐by‐layer film coating and investigate their layer‐dependent and wavelength‐dependent NOA properties. The results of our *Z*‐scan measurements at ≈800 nm show that the MXene films exhibit a strong layer‐dependent NOA behavior. As the layer number increases from 5 to 30, the films transition from RSA to SA, accompanied by a noteworthy variation in the nonlinear absorption coefficient *β*, ranging from ≈7.13 × 10^2^ cm GW^−1^ to ≈−2.69 × 10^2^ cm GW^−1^. In addition, we characterize the NOA of the MXene films at varying incident laser intensities, finding that the *β* decreases as the laser intensity increases. Finally, we characterize the NOA of 2D MXene films at ≈1550 nm by integrating them onto Si_3_N_4_ waveguides. In contrast to the RSA observed for the 5‐layer and 10‐layer films at ≈800 nm, an SA behavior is observed at ≈1550 nm. These results reveal the interesting layer‐dependent nonlinear optical properties of 2D MXene films, which will facilitate the implementation of high‐performance MXene‐based nonlinear photonic devices.

## Conflict of Interest

The authors declare no conflict of interest.

## Author Contributions

D.J., W.L, and L.J. contributed equally to this work. J.W. conceived of the idea and designed the research. W.L performed the MXene synthesis and film coating. D.J., L.J., and J.H. performed material characterization. D.J. and L.J. performed *Z*‐scan measurements. H.D., S.K., C.S., P.D., C.G., and C.M. designed and fabricated the Si_3_N_4_ devices. D.J. and Y.Z. performed data processing and prepared the figures and tables of the manuscript. D.J., J.W., and D.J.M. prepared the text of the manuscript. J.W., D.H., B.J., and D.J.M. jointly supervised the project. All authors participated in the review and discussion of the manuscript.

## Data Availability

The data that support the findings of this study are available from the corresponding author upon reasonable request.
